# Assessing trauma-related symptomatology in the Netherlands: the Dutch Global Psychotrauma Screen (GPS)

**DOI:** 10.1080/20008066.2025.2572850

**Published:** 2025-10-31

**Authors:** Federica Nava, Birit F. P. Broekman, Miranda Olff, Chris M. Hoeboer

**Affiliations:** a Department of Psychiatry, Amsterdam UMC Location University of Amsterdam, Amsterdam, The Netherlands; b Amsterdam Public Health, Mental Health, Amsterdam, The Netherlands; c Department of Psychiatry and Psychological Medicine, OLVG, Amsterdam, The Netherlands; d ARQ National Psychotrauma Centre, Diemen, The Netherlands

**Keywords:** Trauma-related disorders, posttraumatic stress disorder, validation study, screening test, Global Psychotrauma Screen, Trastornos relacionados con el trauma, trastorno de estrés postraumático, estudio de validación, test de mapeo, Mapeo del Psicotrauma global

## Abstract

**Background:** Most adults have experienced at least one Potentially Traumatic Event (PTE) in their life. Yet, many individuals who develop adverse outcomes such as posttraumatic stress disorder (PTSD) are not identified and therefore not treated. Hence, there is a need for better screening tools for trauma-related disorders after exposure to PTEs.

**Objective:** The aim of the present study was to assess validity and reliability of the Dutch version of the Global Psychotrauma Screen (GPS), an easy-to-administer transdiagnostic screener for trauma-related disorders, using a community-based sample of the Dutch adult (16+) population.

**Method:** An online survey was conducted among 1377 Dutch adults. Clinical semi-structured interviews were used as gold-standard comparative measures of PTSD (CAPS-5), depression, and anxiety (MINI) in a subset of participants (*n* = 188). The psychometric characteristics of the Dutch GPS were assessed. We determined the optimal cutoffs for screening for (complex) PTSD, anxiety and depression. We compared the Dutch GPS screening performance with other established screeners (PCL-5, GAD-7, and PHQ-9). We performed regression analyses and analyses of covariance to identify risk-factors and symptoms severity groups regarding impaired social and work functioning.

**Results:** The Dutch GPS showed good performance regarding internal consistency, convergent, and divergent validity, and excellent screening performance for (complex) PTSD, anxiety, and depression comparable to screeners for these specific disorders. Younger age, female sex, being unemployed, having a history of mental illness, having experienced childhood trauma, or other stressful events, and low social support were risk factors for higher GPS total symptom score.

**Conclusions:** The Dutch GPS is a valid and reliable instrument for detection of PTSD and other trauma-related disorders. It is therefore a valuable easily applicable tool that can support diagnostic processes in both research and clinical practice across a wide variety of settings.

## HIGHLIGHTS

- The Dutch GPS is a reliable and valid instrument to detect trauma-related disorders, with a screening performance comparable to other screeners (PCL-5, GAD-7, and PHQ-9).
- Optimal cutoffs were found for the diagnoses of PTSD (3), CPTSD (4), GAD (1), and MDD (1 or 2).
- Younger age, female sex, being unemployed, having a history of mental illness, having experienced childhood trauma, other stressful events, and low social support were identified as risk factors for higher GPS total symptom score.

## Abstract (es)

**Antecedentes:** La mayoría de los adultos han experimentado por lo menos un Evento Potencialmente Traumático (EPT) en su vida. Sin embargo, muchos individuos que desarrollan resultados adversos, tales como trastorno de estrés postraumático (TEPT) no son identificados y, por lo tanto, no son tratados. Por eso, existe una necesidad de mejores herramientas de mapeo para los trastornos relacionados con el trauma luego de una exposición a EPT.

**Objetivo:** El objetivo del presente estudio fue evaluar la validez y confiabilidad de la versión holandesa del Mapeo de Psicotrauma Global (GPS en su sigla en inglés), una herramienta de mapeo transdiagnóstica para los trastornos relacionados con el trauma fácil de administrar, usando una muestra comunitaria de la población adulta holandesa (16 años y más).

**Método:** Una encuesta en línea fue administrada a 1377 adultos holandeses. Entrevistas clínicas semi-estructuradas se usaron como medidas comparativas de excelencia para TEPT (CAPS-5), depresión y ansiedad (MINI) en una muestra de participantes (*n* = 188). Se evaluaron las características psicométricas del GPS holandés. Determinamos los puntajes de corte óptimos para el mapeo de TEPT (complejo), ansiedad, y depresión. Comparamos el desempeño de la capacidad de detección del GPS holandés con las otras medidas de mapeo establecidas (PCL-5, GAD-7 and PHQ-9). Realizamos análisis de regresión y análisis de covarianza para identificar los grupos de factores de riesgo y los síntomas de severidad con respecto al funcionamiento social y de trabajo afectados.

**Resultados:** El GPS holandés mostro un buen desempeño con respecto a la consistencia interna, validez convergente y divergente, y un desempeño de mapeo excelente para TEPT (complejo), ansiedad y depresión, comparable con las herramientas de mapeo para estos trastornos específicos. Los factores de riesgo para un puntaje total más alto en el GPS fueron edad más joven, sexo femenino, estar desempleado, tener una historia de trastorno mental, haber experimentado trauma infantil, u otros eventos estresantes, y bajo apoyo social.

**Conclusiones:** El GPS holandés es un instrumento válido y confiable para la detección del TEPT y de otros trastornos relacionados al trauma. Por lo tanto, es una herramienta valorable fácil de aplicar que puede apoyar el diagnóstico realizado tanto en investigación como en la práctica clínica a lo largo de una variedad de contextos.

## 1. Introduction

The majority of the world’s population experiences at least one Potentially Traumatic Event (PTE) (Kessler et al., 2017; Schnyder et al., 2017). In the Netherlands, recent findings show that about 81.5% of the adult population has been exposed to at least one kind of PTE during their life (Hoeboer et al., 2025). Furthermore, a significant treatment gap was identified, with about two-thirds of those with posttraumatic stress disorder (PTSD) not receiving care (Hoeboer et al., 2025). This highlights the need for improved screening to identify those with mental health problems, and the need for the development of easily accessible preventative interventions, especially in at-risk populations or after PTEs with a high chance of eliciting mental health problems, like sexual and physical violence (Hoeboer et al., 2025). Consequences of exposure to PTEs range from the well-documented PTSD and complex PTSD (CPTSD), to other trauma-related conditions, such as generalized anxiety disorder (GAD), major depressive disorder (MDD), and substance abuse (Goldstein et al., 2016). These trauma-related disorders frequently co-occur (Spinhoven et al., 2014), and the transdiagnostic nature of the consequences of exposure to PTEs has been widely demonstrated (Frewen et al., 2021). The co-occurrence of disorders results in a great negative impact on people’s quality of life (Pagotto et al., 2015). Nevertheless, not everyone exposed to PTEs develops trauma-related disorders, meaning that the presence of risk and protective factors might influence the symptom trajectory (Brewin et al., 2000). Therefore, the scientific community has been addressing the need of detecting risk and protective factors in the development of trauma-related disorders (Dietrich, 2001; Hettema et al., 2005; Hölzel et al., 2011; Kessler et al., 2017), resulting in several well-established risk and protective factors. Female sex, childhood trauma, and prolonged trauma have been associated with increased risk, whereas social support and resilience appear to reduce the likelihood of developing a trauma-related condition (Olff et al., 2025; Tortella-Feliu et al., 2019). This information can be used for (targeted) screening and interventions.

Several screening instruments exist to evaluate PTSD (Mouthaan et al., 2014). Mouthaan et al. (2014) compared three PTSD screening instruments and found that despite differences in length (ranging from 4 to 22 items), all instruments showed similar adequate accuracy, modest specificities, and low positive predictive values. Moreover, these screeners were all limited to PTSD. In fact, existing screeners for most mental health disorders tend to focus on only one specific disorder, ignoring the transdiagnostic impact of trauma exposure, and increasing the time required to reach a complete picture of possible symptomatology (Frewen et al., 2021). In order to identify persons at risk of developing trauma-related symptomatology, a more comprehensive e transdiagnostic screener that takes risk and protective factors into account is needed.

As part of the Global Collaboration on traumatic stress (GCTS) (Schnyder et al., 2017) the Global Psychotrauma Screen (GPS) was developed to identify different potential trauma-related outcomes, as well as risk and protective factors (Olff et al., 2020). The aim of the group was to create an instrument easy to administer, cross-culturally valid and free of charge (Olff et al., 2020). The GPS is a short screener that allows detection of trauma-related symptomatology, such as (C)PTSD, GAD, MDD, substance abuse, as well as risk and protective factors. It consists of 22 items with dichotomous answer scales. The GPS has been translated in over 30 languages, following the translation procedure described by Sousa and Rojjanasrirat (2011) or similar methods. Various validation studies on the psychometric characteristics of the GPS showed good reliability and validity of the instrument in Indonesian, Iranian, US English, Canadian English, Japanese, Greek, Italian, and Brazilian populations (Brunnet et al., 2025; Frewen et al., 2021; Grace et al., 2023; Koutsopoulou et al., 2024; Oe et al., 2020; Primasari et al., 2024; Rossi et al., 2020; Salimi et al., 2023).

The aim of the current study was to validate the Dutch GPS, using a community-based sample of the Dutch population, which included both adolescents (16+) and adults. Clinical semi-structured interviews were used as gold-standard comparison measures for PTSD, MDD, and GAD. Specific objectives included the assessment of the internal consistency, construct, convergent, divergent, and clinical validity of the Dutch GPS, as well as the analysis of the relationship between risk factors, trauma-related symptoms, and social/work functioning. Moreover, we also aimed to compare the screening performance of the GPS with established screeners for the specific disorders.

## 2. Materials and methods

### 2.1. Design and participants

The data for this study were collected as part of a larger cross-sectional study, aimed at determining the prevalence of PTSD in Dutch adults (Hoeboer et al., 2025). The study was reviewed by the Medical Ethics Review Committee of Amsterdam University Medical Centers (Ref.: FWA00032965), which determined that it did not fall under the scope of the Medical Research Involving Human Subjects Act (WMO).

Recruitment and sampling were carried out by the Longitudinal Internet studies for the Social Sciences (LISS) panel (CenterData, 2024). The LISS database is an online stratified panel, consisting of a random selection of households, based on census data (CBS, 2024). A detailed description of the sampling procedure used by the panel can be found elsewhere (Hoeboer et al., 2025). Inclusion criteria were being 16 years or older, being able to speak and read Dutch, and living in the Netherlands.

### 2.2. Procedure and sample size

Of the 1947 randomly selected individuals from the LISS database, 1581 (81.2%) responded and completed the measures relevant for the present study. From these, only trauma-exposed participants were included (*n* = 1268). Note that students were included in the study through a separate parallel study on trauma and PTSD in Dutch students. From this parallel study, 130 participants were randomly selected to represent the student share in the Dutch general population. Of these, only trauma-exposed students were included (*n* = 109). The final database was therefore composed of 1377 participants.

Participants completed online questionnaires between 1st of September 2023 and 1st of November 2023. A subset of participants voluntarily agreed to a follow up interview (*n* = 188), consisting of two structured clinical interviews assessing trauma-related symptomatology. This subset did not include students due to practical reasons. A researcher with a master’s degree in psychology performed the interviews, either online or via telephone. Interviews were recorded to evaluate inter-rater reliability.

### 2.3. Measures

#### 2.3.1 Demographics

The LISS panel contains demographic information about the participants. For the purpose of this study, baseline information regarding sex, marital status, educational level, primary occupation, cultural background, and urban character of place of residence were extracted.

#### 2.3.2 PTEs

The Dutch version of the Life Events Checklist for DSM-5 (LEC-5) (Weathers et al., 2013) was used to assess lifetime exposure to PTEs. The LEC-5 is a 16-item checklist, listing 16 types of PTEs for which participants indicate different ways of exposure (self-experienced, experienced as a witness, happened to a family member or a close friend, experienced as part of one’s job). The LEC-5 PTEs were grouped into two categories: interpersonal PTEs (items 6, 7, 8, 9, 10, 11, 16) and non-interpersonal PTEs (items 1, 2, 3, 4, 5, 12, 13, 14, 15).

#### 2.3.3 GPS

The Dutch version of the Global Psychotrauma Screen (GPS, https://www.global-psychotrauma.net/gps) (Olff et al., 2020) was used to assess current PTSD. The GPS is a 23-item screener for trauma-related symptomatology. The first 22 items are dichotomous and divided into the following subdomains: PTSD (5 items), disturbances in self-organization (DSO) (2 items), anxiety (2 items), depression (2 items), insomnia (1 item), self-harm (1 item), dissociation (2 items), substance abuse (1 item), other physical, emotional or social problems (1 item), and risk and protective factors (5 items: childhood trauma, past mental illnesses, other stressful events, social support, and psychological resilience). Subdomain scores can be obtained by summing the relevant items, providing an indication of the presence and severity of each condition. In addition, a CPTSD score can be calculated by combining the subdomain scores for PTSD and DSO. An additional 23rd item investigates current functioning on a 10 point scale. Participants were asked to report complaints in the past month, referring to an index trauma event. A total symptom score can be calculated by summing up the single item scores of symptom items 1–16 and 18. The total symptom score does not refer to a specific single condition or diagnosis, but to the general severity or overall burden of trauma-related outcomes (range 0–17 with higher scores indicating higher severity/burden). A total risk factor score can be calculated by summing up the single scores of the 5 items regarding risk and protective factors (resilience item reversely coded). The GPS has shown good psychometric properties in previous validation studies (e.g. Frewen et al., 2021; Salimi et al., 2023).

#### 2.3.4 DSM-5 PTSD

The Dutch version of the PTSD Checklist for DSM-5 (PCL-5) (Blevins et al., 2015; Boeschoten et al., 2014) was used to assess current PTSD. Participants were asked to report complaints in the past month, referring to an index trauma event. The PCL-5 is a 20-item questionnaire with 5-point Likert scales. As an additional measure of PTSD, a subset of volunteers was interviewed with the Dutch version of the Clinician-Administered PTSD Scale for DSM-5 (CAPS-5) (Weathers et al., 2018). The CAPS-5 is a 20-item interview with 5-point Likert scales. Both PTSD instruments are based on the four DSM-5 clusters necessary to formulate a PTSD diagnosis: intrusive symptoms, persistent avoidance of thoughts, feelings or places related to the PTE, negative alterations in cognitions and mood, and marked alteration in arousal and reactivity (APA, 2013). A total score is calculated by summing up the single item’s scores (both instruments range 0–80 where higher scores indicate higher severity of the reported symptomatology). For a PTSD diagnosis according to the CAPS-5 the following requirements need to be satisfied: at least one item of the intrusive symptoms cluster needs to score 2 or higher; at least one item of the avoidance cluster needs to score 2 or higher; at least two items of the negative alterations cluster need to score 2 or higher; at least two items of the alteration in arousal cluster need to score 2 or higher; the disturbance has lasted for at least one month; presence of significant distress or functional impairment related to the disturbances. Both the Dutch PCL-5 and CAPS-5 showed good psychometric properties (Boeschoten et al., 2018; Hoeboer et al., 2024). In the current study, Cronbach’s alpha revealed high internal consistency (.953). Inter-rater reliability was calculated on 10% of the CAPS-5 interviews. A second researcher listened to the recordings of the interview and scored the CAPS-5 independent of the original interviewer. Cohen’s kappa showed high agreement between the two researchers (kappa = .87).

#### 2.3.5 ICD-11 CPTSD

With reference to the past month and the same index trauma event as the GPS, PCL-5, and CAPS-5, participants completed the Dutch version of the International Trauma Questionnaire (ITQ) (Cloitre et al., 2018). The ITQ is an 18-item self-report questionnaire using 5-point Likert scales assessing CPTSD following the ICD-11 criteria (scored 0–10 with higher scores indicating higher symptom severity). It is divided according to ICD-11 symptom criteria into two major subscales: PTSD (six items) and DSO (six items). The PTSD major subscale includes: intrusions, avoidance, and sense of threat. The DSO major subscale includes: affective dysregulation, negative self-concept, and disturbances in relationships. Additionally, functional impairment associated with PTSD symptoms and DSO is assessed (six items). For a CPTSD diagnosis according to the ITQ the following requirements need to be satisfied: at least one item per symptom cluster in the PTSD subscale has to be scored ‘moderate’ or higher, at least one item has to indicate functional impairment due to PTSD symptoms, at least one item per symptom cluster in the DSO subscale needs a score of ‘moderate’ or higher, and at least one item has to indicate functional impairment related to DSO. Good psychometric characteristics were reported in the Dutch ITQ (Gerrmann et al., 2023).

#### 2.3.6 Other trauma-related outcomes

The Dutch version of the General Anxiety Disorder-7 (GAD-7) (Spitzer et al., 2006) questionnaire was used to assess GAD symptomatology and severity of symptoms in the past two weeks. The GAD-7 is a 7-item self-report screening questionnaire with 4-point Likert scales. A total score is calculated as the sum of the single items’ scores (range 0–21 with higher scores indicating higher symptom severity). The Dutch version of the GAD-7 showed good psychometric qualities (Donker et al., 2011). In the current study, Cronbach’s alpha revealed high internal consistency (.920).

The Dutch version of the Patient Health Questionnaire-9 (PHQ-9) (Kroenke et al., 2001) was used to assess MDD in the past two weeks. The PHQ-9 is a 9-item self-report screening questionnaire with 4-point Likert scales reflecting the DSM-5 symptoms of depression (range 0–27 with higher scores indicating higher symptom severity). The Dutch version of the PHQ-9 showed good internal consistency and convergent validity (Kroenke et al., 2001). In the current study, Cronbach’s alpha revealed high internal consistency (.899).

The Dutch version of the Alcohol, Smoking and Substance Involvement Screening Test (ASSIST) (Group WHO ASSIST Working, 2002) was used to identify substance abuse related health risks and substance abuse disorders. The ASSIST is a interview-based questionnaire with 5-points Likert scales (range 0–39 with higher scores indicating higher symptom severity) and it consists of two parts: in the first section participants were asked about their lifetime use of substances; the second section contained questions about the past three months relating to the substance use, regarding the frequency of use, urge to use, health, social, judicial or financial problems, failure to perform expectations, concern of external people over the use of substances, impossibility to control the use of substances, and use by injection. The ASSIST has shown good psychometric properties in multiple languages (Group WHO ASSIST Working, 2002).

As an additional measure of MDD, GAD, and substance abuse, the Dutch version of the Mini-International Neuropsychiatric Interview (MINI, version 5.0.0) (Sheehan et al., 1998) was also administered during the interviews. The MINI is a short, structured interview based on DSM-IV and ICD-10 diagnostic criteria for all main psychiatric disorders. It is composed by different sections, where specific instructions lead the interviewer to different sets of questions depending on the respondent’s answers. For the purpose of this study, the sections regarding MDD, GAD, and substance abuse were included.

### 2.4. Statistical analyses

A detailed description of the data-analysis plan was preregistered at the open science framework (https://doi.org/10.17605/OSF.IO/V8NCF) (Nava et al., 2025). Analyses were performed with IBM SPSS Statistics Version 28.0 and R version 3.6.1. Alpha was set to .05 for all analyses (two-sided), to allow for the possibility of effects in both positive and negative directions. All assumptions of the analyses for factor analyses (linearity, data level, sample size, outliers, multicollinearity, common variance, multivariate normality, relevance of variables, independence of factors), correlation (level of measurement, related pairs, absence of outliers, linearity), regression (linearity, normality, multicollinearity), and analysis of covariance (normality, homogeneity of variances, homogeneity of regression slopes, a linear relationship between the dependent variable and covariate, independence of observations, outliers analysis) were checked and analyses were subsequently adapted when assumptions were violated. Since assumptions for some analyses were not met, non-parametric tests were chosen for correlation (Spearman’s correlation), regression (hierarchical multiple regression analysis with bootstrapping), and covariance (covariance analysis with bootstrapping) analyses. Firstly, descriptive analyses were performed to describe the sample according to baseline characteristics. Descriptive statistics were also used to describe exposure to different kinds of PTEs and to non-interpersonal trauma and interpersonal trauma.

#### 2.4.1 Factor analysis

The factor structure of the Dutch GPS was explored with Exploratory Factor Analysis (EFA) and Confirmatory Factor Analysis (CFA). See Appendix A for methods and results of these analyses.

#### 2.4.2 Internal consistency and validity analyses

Internal consistency analyses were performed using Cronbach’s Alpha, inter-item correlation, item-total correlation, and Cronbach’s Alpha with item deletion. Convergent validity was assessed for the GPS total symptom score, by using other measures of the different subdomains included in the GPS total symptom score. Spearman’s Correlation coefficient was used between GPS total symptom score and PCL-5, ITQ, GAD-7, PHQ-9, ASISST, Self-harm according to PHQ-9, CAPS-5 Dissociation score, and CAPS-5 impaired social and work functioning. Spearman Correlation analyses were also performed to test the convergent and divergent validity of the Dutch GPS for GPS subdomain scores. Every GPS subdomain score was correlated with either parallel measures of the same construct (e.g. GPS PTSD with PCL-5) or parallel measures of the other subdomains’ constructs (e.g. GPS PTSD with PHQ-9). Furthermore, clinical validity was assessed to determine AUC, sensitivity, specificity, positive predictive value (PPV), and negative predictive value (NPV) of the Dutch GPS. Optimal cutoff points were calculated for DSM-5 PTSD, ICD-11 CPTSD, MDD, GAD, and alcohol abuse using Youden index, to maximize sensitivity and specificity. The optimal cutoff points of DSM-5 PTSD, MDD, GAD, and alcohol abuse were determined using CAPS-5 and MINI (MDD, GAD, and AUD) scores as gold standard references. The optimal cutoff point of ICD-11 CPTSD was calculated using the ITQ score as gold standard reference. Finally, additional clinical validity analyses were performed to compare the screening performance of the GPS versus the PCL-5, PHQ-9, and GAD-7. For these instruments, two types of cutoffs were used. A first one was calculated based on the current sample using Youden index and the CAPS-5 (for PCL-5) or MINI (for PHQ-9 and GAD-7) as gold standard measures. An additional cutoff was considered for the PCL-5, PHQ-9, and GAD-7 from previous literature (Donker et al., 2011; Hoeboer et al., 2024; Kroenke et al., 2001). For the PCL-5 a cutoff of 22 was added to the analyses (Hoeboer et al., 2024), for the PHQ-9 (Kroenke et al., 2001) a cutoff of 5, and for the GAD-7 a cutoff of 12 (Donker et al., 2011).

#### 2.4.3 Risk factors analysis and trauma-related symptoms severity analysis

Hierarchical multiple regression with bootstrapping was used to assess the relationship between risk factors and overall burden of trauma-related outcomes, using the GPS total symptom score. In the first block, sociodemographic variables (sex, age, marital status, educational level, primary occupation, cultural background, and urban character of place of residence) were entered in the model. In the second block, risk factors were entered (other stressful events, low social support, childhood trauma, history of mental illness, low resilience).

Analysis of Covariance (ANCOVA) test with bootstrapping was used to evaluate differences in impaired social/work functioning in different GPS symptom severity groups. The severity groups are defined as low, mild, moderate, and severe according to the following cutoffs: 30th percentile, 75th percentile, 95th percentile (Fissette et al., 2014). Sex, age, marital status, educational level, primary occupation, cultural background, and urban character of place of residence were also included in the ANCOVA as covariates.

## 3. Results

### 3.1. Baseline characteristics

**Figure 1.**
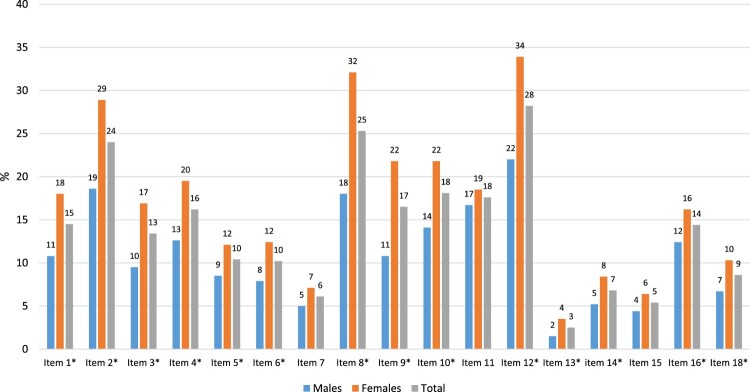
Sex differences in the endorsement of GPS symptom items. * = a statistically significant difference was found.

Table 1 summarizes the baseline characteristics of the participants (*N* = 1377). Females scored significantly higher on the GPS total symptom score (*M* = 2.9, *SD* = 3.8) compared to males (*M* = 1.9, *SD* = 3.2, *t*(1364) = −5.488, *p* < .001). Furthermore, females scored significantly higher on all GPS subdomains, PTSD: *t*(1365) = −5.174, *p* < .001, CPTSD: *t*(1364) = −4.994, *p* < .001, DSO: *t*(1351) = −2.761, *p* = .006, anxiety: *t*(1332) = −6.696, *p* < .001, depression: *t*(1374) = −2.633, *p* = .009, insomnia: *t*(1371) = −4.980, *p* < .001, self-harm: *t*(1255) = −2.363, *p* = .018, dissociation: *t*(1349) = −2.400, *p* = .017, and substance abuse: *t*(1357) = −2.443, *p* = .015. See individual item endorsement for males and females in Figure 1.

**Table 1.** Baseline characteristics of the participants (*N* = 1377).

	No. (%)
Age, mean (SD), years	54.04 (18.50)
PTE, mean (SD)	5.8 (4.4)
Female sex	717 (52.1)
Marital status	
Married	694 (50.4)
Divorced	171 (12.4)
Widow or widower	74 (5.4)
Never married	438 (31.8)
Urban character of place of residence	
Extremely urban	277 (20.1)
Very urban	393 (28.5)
Moderately urban	280 (20.3)
Slightly urban	240 (17.4)
Not urban	182 (13.2)
Missing	5 (0.4)
Education	
Elementary education	79 (5.7)
Pre-vocational secondary education	200 (14.5)
Higher secondary education	171 (12.4)
Secondary vocational education	330 (24.0)
Higher vocational education	395 (28.7)
University	198 (14.4)
Missing	4 (0.3)
Primary occupation	
Employed	605 (43.9)
Freelancer	68 (4.9)
Retired	399 (29.0)
Student	110 (8.0)
Incapacitated	50 (3.6)
Stay-at-home parent/spouse	93 (6.8)
Unemployed	42 (3.1)
Other/Missing	10 (0.7)
Cultural background	
Dutch	1128 (81.9)
First/second generation western	105 (7.6)
First/second generation non-western	114 (8.3)
Missing	30 (2.2)
PTE exposure	
Interpersonal violence	789 (57.3)
Non-interpersonal violence	1285 (93.3)
Number events experienced, mean (SD)	5.79 (4.36)

Note: PTE: Potentially traumatic event.

### 3.2. Internal consistency

GPS total symptom score showed good internal consistency (Cronbach’s alpha = .899), without significant improvement when an item was deleted (Cronbach’s alpha range = .888–.899), showing that all items are contributing consistently and none reduce the overall internal consistency of the scale. Inter-item and item-total correlations were acceptable (respective ranges: .169–.560; .377–.686). The lowest inter-item correlation (.169) was found between item 1 (nightmares) and item 13 (self-harm). The lowest item-total correlation (.377) was found for item 13 (self-harm).

### 3.3. Convergent and divergent validity

GPS total symptom score was significantly positively correlated with other measures of (C)PTSD, GAD, MDD and alcohol and substance abuse (see Appendix B for correlation matrix). GPS subdomains strongly correlated with corresponding predictor variable (e.g. GPS PTSD vs PCL-5). The GPS subdomains showed low correlations with measures of other subdomains (e.g. GPS Anxiety vs ASSIST).

### 3.4. Clinical validity

We were unable to test the screening ability of the GPS for substance abuse disorders, because none of the interviewed participants met criteria for current DSM-5 substance abuse disorder.

Regarding the GPS, we found a cutoff of 3 to be optimal for detection of PTSD, a cutoff of 4 for CPTSD, and a cutoff of 1 for GAD. For MDD, two cutoffs (1 and 2) are presented since they perform almost equally well and the choice between them depend on the relative emphasis on sensitivity versus specificity. Regarding other instruments, a cutoff of 37 was found to be optimal for PCL-5 according to the CAPS-5, and a cutoff of 7 was found to be optimal for both GAD-7 and PHQ-9 according to the MINI.

Table 2 summarizes the results regarding the performance of the GPS in detecting clinical diagnoses. GPS subdomain PTSD showed excellent screening performance (in terms of AUC, sensitivity, and specificity), followed by a good performance of GPS subdomains CPTSD, MDD, and GAD. The screening ability of the GPS was highly comparable to the PCL-5 for PTSD and GAD-7 for GAD, but the performance of the GPS for detecting MDD was substantially lower compared to the PHQ-9 especially for sensitivity.

**Table 2.** Clinical validity of Dutch GPS subdomain scores and other screening measures of PTSD, GAD, and MDD versus CAPS-5 PTSD, MINI anxiety, and MINI depression.

Subdomains	Cutoff (sample-based)	Cutoff (literature-based)	Youden’s index	Sensitivity	Specificity	PPV	NPV	AUC
GPS PTSD	3		.85	1.00	.85	.16	1.00	.94
PCL-5 PTSD	37		.93	1.00	.93	.29	1.00	.98
PCL-5 PTSD		22	.83	1.00	.83	.15	1.00	.98
GPS CPTSD	4		.72	.83	.89	.21	.99	.84
GPS ANX	1		.52	.82	.70	.22	.97	.79
GAD-7 ANX	7		.67	.76	.91	.46	.97	.91
GAD-7 ANX		12	.50	.53	.97	.64	.95	.91
GPS DEPR	2		.59	.71	.87	.32	.97	.81
GPS DEPR	1		.52	.79	.73	.20	.98	.81
PHQ-9 DEPR	7		.79	.93	.86	.36	.99	.90
PHQ-9 DEPR		5	.71	.93	.78	.27	.99	.90

Note: PPV: Positive Predicted Value; NVP: Negative Predicted Value; AUC: Area Under the Curve.

### 3.5. Risk factors

Table 3 shows the results of the bootstrapped hierarchical multiple regression analysis. In the first step of the model with only demographics, 7.1% of the variance was explained (*F*(7,1329) = 14.440, *p* < .001). In the second step of the model including GPS risk factors, 45.1% of the variance was explained (*F*(12,1324) = 90.624, *p* < .001). We found that younger age (*p* = .002), female sex (*p* < .001), being unemployed (*p* = .002), experiencing other stressful events (*p* < .001), low social support (*p* < .001), having a history of mental illness (*p* < .001), and having experienced childhood trauma (*p* < .001) were related to higher GPS total symptom score. We found no significant association between GPS total symptom scores and marital status, educational level, cultural background, urban character of the place of residence, and resilience.

**Table 3.** Risk factors of Dutch GPS total symptom score.

Risk factors	t	B	Bootstrap (1000 samples)			
			B 95% CI		SE	*p*-value
Age	−6.173	−.037	−.051	−.023	.007	<.001**
Sex	3.808	.727	.361	1.109	.187	<.001**
Marital status	−2.081	−.421	−.804	−.007	.193	.031*
Educational level	.053	.011	−.422	.484	.217	.952
Cultural background	1.464	.381	−.236	.996	.314	.216
Primary occupation	−5.150	−1.117	−1.571	−.631	.232	<.001**
Urban character	−.232	−.048	−.456	.379	.207	.826
Age	−3.383	−.016	−.026	−.006	.005	.006**
Sex	3.444	.508	.205	.814	.147	.002**
Marital status	−.029	−.005	−.316	.309	.155	.974
Education	.141	.023	−.276	.329	.161	.888
Cultural background	.768	.155	−.264	.552	.207	.447
Primary occupation	−2.993	−.496	−.853	−.133	.175	.003**
Urban character	−.309	−.049	−.356	.300	.166	.772
Other stressful events	12.892	3.300	2.616	4.047	.350	<.001**
Low social support	13.580	3.357	2.710	4.008	.365	<.001**
History mental illness	10.430	2.060	1.536	2.524	.266	<.001**
Childhood trauma	4.938	.817	.419	1.193	.190	<.001**
Resilience	1.315	.301	−.157	.767	.247	.218

Note. B 95% CI = Bias-corrected and accelerated Confidence Interval; SE = Standard Error; **. *P*-value is significant at the 0.01 level (2-tailed); *. *P*-value is significant at the .05 level (2-tailed).

### 3.6. Trauma-related symptoms severity

Bootstrapped ANCOVA analyses showed a significant difference in the four symptoms severity groups regarding impaired social and work functioning (*F*(3, 169) = 16.59, *p* *<* .001), while adjusting for demographic variables. Furthermore, higher level of impairment was found in more severe symptoms severity groups (*M_1_* = .080, *95% CI* = .027–.140; *M_2_* = .088, *95% CI* = .005–.187; *M_3_* = .348, *95% CI* = .175–.539; *M_4_* = 1.076, *95% CI* = .578–1.592). Specifically, the highest severity group showed the highest level of social and work functioning impairment. When looking at pairwise comparisons, no significant difference was found between the first two severity groups, while a significant difference was found among all other groups.

## 4. Discussion

The present study aimed to validate the Dutch version of the GPS in a community-based sample of the Dutch adult (>16 years) population. Findings of the current study demonstrate that the Dutch GPS is a reliable and valid instrument to assess and screen for PTSD and trauma-related symptomatology in the Dutch general population, comparable to existing disorder-specific screeners. Together with its easy administration and transdiagnostic scope this makes the GPS an attractive alternative to assess a broad range of trauma-related symptomatology.

Internal consistency results suggest that all items of the GPS are related to each other. Moreover, convergent and divergent validity analyses showed that the Dutch GPS reflects the intended construct of trauma-related symptomatology: positive correlations were found between the GPS total symptom score and other diagnostic measures of trauma-related symptoms, while low correlations were observed between GPS subdomains and measures related to different subdomains. This means that the Dutch GPS is a reliable instrument for evaluation of trauma-related symptomatology.

With regard to the GPS subdomains, the Dutch GPS showed good to excellent clinical validity, and proved to be an appropriate screener for PTSD, performing highly comparable to the PCL-5, with an optimal cutoff of 3. The optimal cutoffs found for GAD and CPTSD were 1 and 4 respectively. Compared to the PCL-5 and GAD-7, the Dutch GPS showed similar sensitivity, specificity, NPV and PPV. This means that all these instruments accurately identify PTSD and GAD. For clinical use, the GPS is particularly well-suited for screening contexts where various trauma-related disorders need to be identified in an efficient way. For example, in the aftermath of a natural traumatic event or in emergency care settings, the Dutch GPS would be optimal to evaluate briefly and efficiently the presence of any trauma-related disorders. Conversely, in settings where only one disorder is relevant or where it is particularly important to accurately identify those without the disorder, longer disorder-specific instruments may be preferable due to their higher specificity. For screening of MDD, two cutoffs were found, 2 and 1, respectively indicating more specific and more sensitive thresholds. The advice for the clinical practice is to use the higher cutoff in situations where true negatives are important, while the lower cutoff is recommended when true positives are to identify. However, for both cutoffs the PHQ-9 performs somewhat better especially in terms of sensitivity. Therefore, the PHQ-9 is preferred when it is important to accurately identify those with MDD, minimizing false positives, while the Dutch GPS can be used in situations where the goal is to identify those without MDD.

We also identified risk factors for higher GPS total symptom scores: younger age, female sex, being unemployed, having a history of mental illness, having experienced childhood trauma, or other stressful events, and low social support. These results are in line with previous GPS-validation studies (Grace et al., 2023; Primasari et al., 2024; Rossi et al., 2020), although lack of resilience was not a risk factor in our study. This could be explained by the fact that our database was population-based, while other studies where resilience was found as a predicting factor were for example composed by students (Primasari et al., 2024), or COVID-19 sample (Rossi et al., 2020).

Finally, greater social and work impairment (CAPS-5) was found in the group with higher symptom severity, which is in accordance with previous literature (Mahoney & Marx, 2022). This confirms that PTSD and trauma-related disorders affect people lives in their whole, constituting therefore a societal issue (Magruder et al., 2017). Prevention and early detection of PTSD and trauma-related conditions is consequently crucial (Hoeboer et al., 2025).

The Dutch GPS is an easy-to-administer, reliable screener, making it suitable for diverse contexts, including post-disaster crisis response situations, emergency care settings, General Practitioners’ practices, and research environments. It offers a valuable balance between speed and accuracy in detecting trauma-related symptoms early after a PTE, allowing for timely interventions to help prevent the onset of PTSD or other trauma-related disorders.

Our study has several limitations. First, we could not evaluate the clinical validity of the alcohol abuse subdomain, because no participants in the interviews met the criteria for current DSM-5 substance abuse disorder. Second, test–retest reliability could not be assessed due to the cross-sectional nature of the present study. Nevertheless, our study also has important strengths. In fact, this is the first study validating the GPS in the Netherlands, using the CAPS-5 and the MINI as clinical reference standards. The use of clinical interviews makes the results more accurate and reliable. Furthermore, the database was composed of a large population-based sample. Finally, this study provides comparison with other screeners and allows clinicians and researchers to choose which tool to use according to the setting and the purpose.

In conclusion, this study demonstrates that the GPS can be used as a valid screener for PTSD and other trauma-related symptomatology in the Netherlands. It is an efficient and broad transdiagnostic screener, easy to administer, freely available through the GCTS website (www.global-psychotrauma.net/gps), that allows evaluation of multiple symptoms in one brief questionnaire. Thanks to these characteristics it can support both research and clinical practice diagnostic processes, facilitating identification of trauma-related conditions in the Dutch population.

## Author contributions

Federica Nava: Data curation, Formal analysis, Writing original draft. Birit F. P. Broekman: Conceptualization, Methodology, Writing review & editing. Miranda Olff: Conceptualization, Funding acquisition, Methodology, Writing review & editing. Chris M. Hoeboer: Conceptualization, Funding acquisition, Methodology, Supervision, Writing review & editing.

## Data Availability

All pseudo anonymous data will be available via the LISS Panel Data Archive: https://www.dataarchive.lissdata.nl.

## Appendices

### EFA and CFA analyses methods

EFA and CFA were performed to determine the factor structure of the Dutch GPS. The database containing the 17 GPS symptom items was split into two subsets, using the Solomon methodology (Lorenzo-Seva, 2022). Two steps of analyses were conducted. Firstly, tetrachoric EFA using oblique rotation methods and principal axis factoring was performed on one subset. The number of factors to retain was investigated through visual inspection of the scree plot, Parallel Analysis (PA), and examination of the eigenvalue of the factors (greater than 1). Factor loadings (greater than .3), the variance explained by the factors, and absence of cross-loading were also investigated.

CFA was performed on the second subset, testing adequate models from the EFA, and one, two, and three factors models that were found in previous validations of the GPS (Brunnet et al., 2025; Frewen et al., 2021; Grace et al., 2023; Koutsopoulou et al., 2024; Primasari et al., 2024; Salimi et al., 2023). The Weighted Least Squares estimator was used, as it is more appropriate for non-continuous data (Sass et al., 2014), and we allowed correlations between factors in the model. The CFA results were evaluated by comparing fit-indices, and good fit was considered as follows: the comparative fit index (CFI) and Tucker – Lewis index (TLI) have to be ≥ .95, the root mean square error of approximation (RMSEA) has to be < .06, and the standardized root mean square residual (SRMR) has to be ≤ .08 (Hu & Bentler, 1999).

### Additional models tested in CFA analyses

#### Grace model (Grace et al., 2023)

PTSD symptoms, self-harm, and substance abuse: items 1, 2, 3, 4, 5, 13, 18;

Dissociative symptoms, anxiety, insomnia, and other problems: items 8, 9, 12, 14, 15, 16;

Negative affect (DSO and depressive symptoms): items 6, 7, 10, 11.

#### Koutsopoulou model (Koutsopoulou et al., 2024)

PTSD, anxiety, and depression: items 2, 3 4, 5, 8, 9, 10, 11, 16;

Altered states of consciousness (depersonalization, derealization, insomnia, substance use, and self-harm): items 1, 12, 13, 14, 15, 18;

DSO: items 6, 7.

#### Salimi model (Salimi et al., 2023)

Negative affect: items 4, 5, 6, 7, 8, 9, 10, 11, 13, 16, 18;

Dissociative symptoms: items 14, 15;

Core PTSD symptoms: items 1, 2, 3, 12.

#### Brunnet model (Brunnet et al., 2025)

Numbing, guilt, worthlessness, worry, depression, and anhedonia: items 4, 5, 6, 9, 10, 11, 14, 15;

Anger, anxiety, self-harm, other problems, and substance abuse: items 7, 8, 13, 16, 18;

Core PTSD symptoms: items 1, 2 3, 12.

### EFA and CFA analyses results

All assumptions for EFA were met (Kaiser Meyer-Olkin = .945; Bartlett *p* < .001). The observation of the scree plot suggested a one-factor solution, while the Parallel Analysis suggested a two-factors solution. See Table A.1 and A.2 for items loadings and variance explained of these two solutions. Both one-factor and two-factors solutions seemed acceptable, considering that factor loadings of all items in the models were above the threshold of .40. In the two-factors model, Factor 1 was represented by negative emotions and dissociative symptoms, while Factor 2 included the PTSD core symptoms and additional anxiety and depression symptoms (see Table A.2 for specific items). In this solution, item 4 (numbing) had a similar loading on both factors (.47 and .48). The one-factor and two-factors solutions explained 60% and 69% respectively of the total variance.

Scaled fit indices of CFA are included in Table A.3. All models showed a good fit. The two-factors model and the Salimi model showed best fit indices. These fit indices were highly comparable, although the Salimi model (Negative affect, Dissociative symptoms, and Core PTSD symptoms) performed slightly better.

**Table A1.** Factor loadings for the one-factor solution model of Dutch GPS.

Item	GPS Item	Factor 1
10	Depression	.89
4	Numbing/detachment	.89
6	Worthlessness	.88
9	Worrying	.88
8	Anxiety	.85
11	Anhedonia	.83
14	Derealization	.82
16	Other problems	.79
13	Self-harm	.77
5	Guilt	.76
15	Depersonalization	.76
18	Substance abuse	.75
2	Avoidance	.75
3	Hypervigilance	.75
12	Insomnia	.72
7	Anger	.71
1	Nightmares	.70
	Proportion variance	.63
	SS loadings	1.78

Note: SS loadings: sum of squared loadings.

**Table A2.** Factor loadings for the two-factors solution model of Dutch GPS.

Item		GPS item		Factor 1	Factor 2
13		Self-harm		**.****94**	−.14
7		Anger		.**90**	−.15
14		Derealization		.**85**	.02
15		Depersonalization		.**75**	.05
6		Worthlessness		.**72**	.21
18		Substance abuse		.**64**	.15
11		Anhedonia		.**63**	.25
10		Depression		.**60**	.34
16		Other problems		.**53**	.31
5		Guilt		.**53**	.29
2		Avoidance		−.07	.**90**
1		Nightmares		−.08	.**85**
8		Anxiety		.21	.**70**
3		Hypervigilance		.13	.**68**
12		Insomnia		.11	.**67**
9		Worrying		.33	.**61**
4		Numbing		.47	.**48**
	SS loadings			6.65	5.02
	Proportion variance			.39	.30
	Cumulative variance			.39	.69
					

Note: SS loadings: sum of squared loadings.

**Table A3.** CFA results.

Model	*p*-value (*X*^2^)	CFI	TLI	RMSEA	SRMR
One-factor	< .001	.991	.989	.029	.061
Two-factors	.001	.993	.992	.026	.057
Grace	< .001	.992	.991	.027	.059
Koutsopoulou	< .001	.991	.989	.030	.060
Salimi	.002	.993	.992	.025	.056
Brunnet	< .001	.992	.991	.027	.058

Note: CFI: Comparative Fit Index; TLI: Tucker – Lewis Index; RMSEA: Root Mean Square Error Approximation; SRMR: Standardized Root Mean Square Residual.

### Appendix B

**Table B1.** Convergent and divergent validity of the Dutch GPS.

	Criterion variable	Predictor variable							
		PCL month	ITQ CPTSD	GAD7	PHQ9	ASSIST	PHQ-9 Self-harm	CAPS – social work	CAPS – Dissociation
	GPS total symtpom score	.701**	.220**	.680**	.626**	.134	.321**	.473**	.327**
GPS – PTSD		.612**	.231**	.629**	.507**	.073	.291**	.454**	.320**
GPS – CPTSD		.624**	.227**	.636**	.524**	.094	.324**	.462**	.330**
GPS – DSO		.521**	.295**	.528**	.505**	.117	.468**	.500**	.401**
GPS – Anxiety		.595**	.247**	.624**	.494**	.065	.297**	.489**	.283**
GPS – Depression		.546**	.248**	.537**	.521**	.092	.386**	.414**	.200**
GPS – Insomnia		.603**	.181*	.497**	.622**	.190*	.272**	.385**	.199**
GPS – Selfharm		.252**	.138	.256**	.252**	−.012	.281**	.310**	.237**
GPS – Dissociation		.359**	.307**	.399**	.338**	−.005	.304**	.225**	.387**
GPS – Substance abuse		.372**	.223**	.388**	.409**	.383**	.391**	.397**	.157*
									

Note: **. Correlation is significant at the .01 level (2-tailed). *. Correlation is significant at the .05 level (2-tailed).
